# Long non-coding RNA HOMER3-AS1 drives hepatocellular carcinoma progression via modulating the behaviors of both tumor cells and macrophages

**DOI:** 10.1038/s41419-021-04309-z

**Published:** 2021-11-23

**Authors:** Jian Pu, Wenchuan Li, Anmin Wang, Ya Zhang, Zebang Qin, Zuoming Xu, Jianchu Wang, Yan Lu, Qianli Tang, Huamei Wei

**Affiliations:** 1 Department of Hepatobiliary Surgery, Affiliated Hospital of Youjiang Medical University for Nationalities, Baise, China; 2 Graduate College of Youjiang Medical University for Nationalities, Baise, China; 3 Department of Pathology, Affiliated Hospital of Youjiang Medical University for Nationalities, Baise, China

**Keywords:** Liver cancer, Oncogenes, Long non-coding RNAs, Translational research

## Abstract

The crosstalk between cancer cells and tumor microenvironment plays critical roles in hepatocellular carcinoma (HCC). The identification of long non-coding RNAs (lncRNAs) mediating the crosstalk might promote the development of new therapeutic strategies against HCC. Here, we identified a lncRNA, HOMER3-AS1, which is over-expressed in HCC and correlated with poor survival of HCC patients. HOMER3-AS1 promoted HCC cellular proliferation, migration, and invasion, and reduced HCC cellular apoptosis. Furthermore, HOMER3-AS1 promoted macrophages recruitment and M2-like polarization. In vivo, HOMER3-AS1 significantly facilitated HCC progression. Mechanism investigations revealed that HOMER3-AS1 activated Wnt/β-catenin signaling via upregulating HOMER3. Functional rescue experiments revealed that HOMER3/Wnt/β-catenin axis mediated the roles of HOMER3-AS1 in promoting HCC cellular malignant phenotypes. Furthermore, colony stimulating factor-1 (CSF-1) was also identified as a critical downstream target of HOMER3-AS1. HOMER3-AS1 increased CSF-1 expression and secretion. Blocking CSF-1 reversed the roles of HOMER3-AS1 in inducing macrophages recruitment and M2 polarization. Furthermore, positive correlations between HOMER3-AS1 and HOMER3 expression, HOMER3-AS1 and CSF-1 expression, and HOMER3-AS1 expression and M2-like macrophages infiltration were found in human HCC tissues. In summary, our findings demonstrated that HOMER3-AS1 drives HCC progression via modulating the behaviors of both tumor cells and macrophages, which are dependent on the activation of HOMER3/Wnt/β-catenin axis and CSF-1, respectively. HOMER3-AS1 might be a promising prognostic and therapeutic target for HCC.

## Introduction

Liver cancer is the sixth most commonly diagnosed malignancy and the third leading cause of malignancy-related death worldwide, with about 905,677 newly diagnosed cases and 830,180 liver cancer-caused deaths [1]. The main subtype of liver cancer is hepatocellular carcinoma (HCC), which accounts for 80%-90% of all liver cancer cases [2]. Although modern multidisciplinary therapies have been developed to fight against HCC, including surgical resection, liver transplantation, ablation, transcatheter arterial chemoembolization (TACE), molecular targeted therapies, and immunotherapy, only modest improvement in prognoses of HCC patients has been achieved [3]. Therefore, identifying molecular alterations in HCC that are responsible for HCC tumorigenesis and progression and developing novel more effective treatments are urgently needed [4–6].

The molecular alterations of HCC are very complex, with multiple genetic and epigenetic aberrations [7–9]. The ENCODE consortium shows that more than 80% of the human genome are transcribed into RNAs, whereas less than 2% of the human genome lastly encodes proteins [10]. Thus, the protein non-coding transcripts are significantly more diverse than protein-coding mRNAs [11–13]. Long non-coding RNAs (lncRNAs) are the most diverse class of non-coding transcripts, which also have diversely regulatory roles in various pathophysiological processes [14–19]. Several lncRNAs have been revealed to play critical roles in HCC [20, 21]. lncRNA GPC3-AS1 was reported to enhance HCC cellular proliferation and migration [22]. MAGI2-AS3 was found to suppress HCC cellular malignant phenotype [23]. CASC9 depletion decreased HCC cellular viability [24]. lncRNA-ATB promoted HCC invasion-metastasis cascade [25].

The progression of HCC is not only regulated by malignant phenotypes of themselves, but also by tumor microenvironment (TME) [26–28]. Immune checkpoints inhibitors which enhance antitumor immune responses of T cells have been approved to treat HCC [29]. Apart from T cells, tumor-associated macrophages (TAMs) are another class of critical components of TME [8, 30]. Increasing evidences revealed that TAMs have M2-like phenotypes and contribute to tumor progression [27, 31]. Increased levels of M2-like TAMs in HCC were also revealed to be correlated with the poor prognosis of HCC patients [32]. Compared with the M2-like phenotype, macrophages can also be polarized to M1-like phenotypes, which frequently show antitumor activities [33, 34]. Although accumulating studies have identified several factors inducing M1 or M2 polarization of macrophages, such as the induction of M2 phenotype by IL-4, IL-10, TGF-β, and colony-stimulating factor 1 (CSF-1), the contributions of lncRNAs to M2 polarization of TAMs are still largely unknown [35, 36].

In this study, we identified a poor prognosis-correlated lncRNA HOMER3-AS1 in HCC via analyzing the cancer genome atlas (TCGA) liver hepatocellular carcinoma (LIHC). The expression and clinical relevance of HOMER3-AS1 were further investigated in our HCC cohort. In vitro and in vivo functional experiments were undertaken to explore the biological roles of HOMER3-AS1 in HCC. The molecular mechanisms underlying the roles of HOMER3-AS1 in HCC were also investigated.

## Materials and methods

### Bioinformatics analyses

The correlation between gene expression and overall survival of HCC patients based on the RNA sequencing expression data of HCC tissues from the cancer genome atlas (TCGA) liver hepatocellular carcinoma (LIHC) project was analyzed by the online *in silico* tool GEPIA (http://gepia.cancer-pku.cn/). Gene expression correlation in HCC tissues based on the TCGA LIHC dataset was calculated by GEPIA (http://gepia.cancer-pku.cn/). The genes which have similar expression patterns with HOMER3-AS1 in HCC based on the TCGA LIHC dataset were searched by GEPIA (http://gepia.cancer-pku.cn/).

### Clinical samples

Sixty-eight pairs of HCC tissues and adjacent noncancerous liver tissues were acquired at the Affiliated Hospital of Youjiang Medical University for Nationalities from HCC patients who received surgical resection with written informed consents. All tissues were diagnosed by two experienced pathologists. The clinicopathological characteristics of these 68 cases were presented in Table 1. This study was performed following the Declaration of Helsinki and approved by the Ethics Committee of Affiliated Hospital of Youjiang Medical University for Nationalities.

**Table 1:** Correlation between HOMER3-AS1 expression levels and clinicopathological characteristics in HCC.

Feature	Number	HOMER3-AS1		χ^2^	*p*-value
		low	high		
Age				1.503	0.220
>50	39	22	17		
≤50	29	12	17		
Gender				0.637	0.425
Male	61	29	32		
Female	7	5	2		
HBs antigen				0.360	0.549
Positive	54	26	28		
Negative	14	8	6		
Liver cirrhosis				0.856	0.355
With	13	8	5		
Without	55	26	29		
BCLC stage				3.886	0.049
0-A	40	24	16		
B-C	28	10	18		
AFP (ng/ml)				6.071	0.014
>20	40	15	25		
≤20	28	19	9		
Differentiation				0.183	0.669
I–II	6	4	2		
III–IV	62	30	32		
Encapsulation				4.798	0.091
Complete	8	6	2		
Not complete	41	22	19		
No	19	6	13		
Microvascular invasion				3.985	0.046
Absent	42	25	17		
Present	26	9	17		

*p*-value was acquired by Pearson chi-square tests.

### Cell cultures and reagents

Human immortalized liver cell lines THLE-2 and THLE-3, and human HCC cell lines SK-HEP-1 and SNU-398 were acquired from American Type Culture Collection (ATCC). Human HCC cell line Huh7 and monocytic THP-1 cells were acquired from the National Collection of Authenticated Cell Cultures of the Chinese Academy of Sciences (Shanghai, China). THLE-2 and THLE-3 cells were cultured using BEGM Bullet Kit (Lonza, Walkersville, MD, USA) following the provided instructions. SK-HEP-1 cells were cultured in Eagle’s Minimum Essential Medium (Invitrogen, Carlsbad, CA USA) added with 10% fetal bovine serum (FBS, Invitrogen). SNU-398 and THP-1 cells were cultured in RPMI 1640 medium (Invitrogen) added with 10% FBS. Huh7 cells were cultured in Dulbecco’s modified Eagle’s medium (Invitrogen) added with 10% FBS. All cells were grown at 37 °C containing 5% CO_2_. The cells were authenticated using STR profiles. All cells were routinely tested as mycoplasma-free. Where indicated, cells were treated with 10 μM ICG-001 (Selleck, Houston, TX, USA), 0.2 µg/mL Anti-CSF-1 (AF216, R&D Systems, Minneapolis, MN, USA), or 100 ng/ml phorbol-12-myristate-13-acetate (PMA, Sigma Aldrich, St. Louis, MO, USA).

### Quantitative real-time polymerase chain reaction (qRT-PCR)

The total RNA from tissues and cells was extracted using TRIzol Reagent (Invitrogen). After quantification by UV-visible spectrophotometry, the RNA was reversely transcribed into complementary DNA (cDNA) using the M-MLV Reverse Transcriptase (Invitrogen) and random primers. qRT-PCR was undertaken using the TB Green Premix Ex Taq II (TaKaRa, Tokyo, Japan) on StepOnePlus Real-Time PCR System (Applied Biosystems, Foster City, CA, USA). The primer sequences were 5’-ACACCTCTTCTGCCTCCTC-3’ (sense) and 5’-TGGCTTACATCTGTTATCCC-3’ (anti-sense) for HOMER3-AS1, 5’-GCCGAGTTTTTCGCACTG-3’ (sense) and 5’-GAATCTCCTGGTCCTTGGTTT-3’ (anti-sense) for HOMER3, 5’-CTTCCCCTACCCTCTCAA-3’ (sense) and 5’-CGATTTCTTCCTCATCTTCT-3’ (anti-sense) for MYC, 5’-ACCGTGCTGTGTGCTGTG-3’ (sense) and 5’-TCTCCTTGAGTTTGGCTTCT-3’ (anti-sense) for MMP7, 5’-CAGTGGCAATGAGGATGAC-3’ (sense) and 5’-AGATGAAGGGAAAGAAGGTG-3’ (anti-sense) for IL-1b, 5’-ATTGAGGTCATGGTGGAT-3’ (sense) and 5’-GGAGAAGTAGGAATGTGGA-3’ (anti-sense) for IL-12b, 5’-CTCAGCCTCTTCTCCTTCCT-3’ (sense) and 5’-CTGGTTATCTCTCAGCTCCAC-3’ (anti-sense) for TNF-α, 5’-GTGGAGCAGGTGAAGAATG-3’ (sense) and 5’-GAAATGGGGGTTGAGGTATC-3’ (anti-sense) for IL-10, 5’-AGCAGAGTTTGGTCAGGG-3’ (sense) and 5’-GGCTTTTTGTGGGGTTTTC-3’ (anti-sense) for CD163, 5’-CGCCAAGTCCAGAACCAT-3’ (sense) and 5’-TCTCAAGCAGACCAGCCT-3’ (anti-sense) for ARG1, 5’-CCCTGCTGTTGTTGGTCTG-3’ (sense) and 5’-GCATTGGGGGTGTTATCTCTG-3’ (anti-sense) for CSF-1, 5’-GTCGGAGTCAACGGATTTG-3’ (sense) and 5’-TGGGTGGAATCATATTGGAA-3’ (anti-sense) for GAPDH. GAPDH was employed as an endogenous control. Relative quantification was calculated by the comparative Ct method.

### Plasmids construction and transfection

HOMER3-AS1 full-length sequences were PCR-amplified with the primers 5’-CCCAAGCTTGGAACCCAGGCCCTTCC-3’ (sense) and 5’-CCGCTCGAGCAAGTTCTGAATTTAAGGCAGG-3’ (anti-sense). The PCR products were further inserted into the Hind III and Xho I sites of pcDNA^TM^3.1(+) Vector (Invitrogen) to yield HOMER3-AS1 overexpression plasmid. Two pairs of cDNA oligonucleotides repressing HOMER3-AS1 were synthesized and inserted into the shRNA lentivirus expressing vector pLV3/H1/GFP&Puro (GenePharma, Shanghai, China). The constructed plasmid was co-transfected with pGag/Pol, pRev, and pVSV-G (GenePharma) into HEK-293FT cells to generate shRNA lentivirus repressing HOMER3-AS1. Scrambled non-targeting shRNA lentivirus were used as negative control (NC). The shRNA oligonucleotide sequences were 5’-GATCCGGCCCATGTTTCCCTAGAGTTTTCAAGAGAAACTCTAGGGAAACATGGGCCTTTTTTG-3’ (sense) and 5’-AATTCAAAAAAGGCCCATGTTTCCCTAGAGTTTCTCTTGAAAACTCTAGGGAAACATGGGCCG-3’ (anti-sense) for shRNA-HOMER3-AS1-1, 5’-GATCCGGAGTTTGATGACTCAGTACTTTCAAGAGAAGTACTGAGTCATCAAACTCCTTTTTTG-3’ (sense) and 5’-AATTCAAAAAAGGAGTTTGATGACTCAGTACTTCTCTTGAAAGTACTGAGTCATCAAACTCCG-3’ (anti-sense) for shRNA-HOMER3-AS1-2, 5’-GATCCGTTCTCCGAACGTGTCACGTTTCAAGAGAACGTGACACGTTCGGAGAACTTTTTTG-3’ (sense) and 5’-AATTCAAAAAAGTTCTCCGAACGTGTCACGTTCTCTTGAAACGTGACACGTTCGGAGAACG-3’ (anti-sense) for shRNA-NC. HOMER3 coding sequences (CDS) were PCR-amplified with the primers 5’-CCCAAGCTTCCCTAGAGCCTGCCCATC-3’ (sense) and 5’-CCGCTCGAGGGAATCGTTCATAGAAAACC-3’ (anti-sense). The PCR products were further inserted into the Hind III and Xho I sites of pcDNA^TM^3.1(+) Vector to yield the HOMER3 overexpression plasmid. One pairs of cDNA oligonucleotides repressing HOMER3 were synthesized and inserted into pLV3/H1/GFP&Puro (GenePharma). The constructed plasmid was co-transfected with pGag/Pol, pRev, and pVSV-G into HEK-293FT cells to generate shRNA lentivirus repressing HOMER3. The shRNA oligonucleotide sequences were 5’-GATCCCAAACCAAGGACCAGGAGATTTTCAAGAGAAATCTCCTGGTCCTTGGTTTGTTTTTTG-3’ (sense) and 5’-AATTCAAAAAACAAACCAAGGACCAGGAGATTTCTCTTGAAAATCTCCTGGTCCTTGGTTTGG-3’ (anti-sense) for shRNA-HOMER3. Plasmids transfection and co-transfection were undertaken with Lipofectamine 3000 (Invitrogen).

### Stable cell lines construction

To construct HOMER3-AS1 stably overexpressed and control cells, HOMER3-AS1 overexpression plasmid or empty plasmid pcDNA3.1 was transfected into THLE-2, SK-HEP-1, and SNU-398 cells. The transfected cells were treated with 800 µg/ml neomycin 48 h after transfection for 4 weeks to select HOMER3-AS1 overexpressed cells. To construct HOMER3-AS1 stably silenced and control cells, SK-HEP-1, and Huh7 cells were infected with shRNA lentivirus repressing HOMER3-AS1 or scrambled non-targeting shRNA lentivirus. The infected cells were treated with 2 µg/ml puromycin 96 h after infection for four weeks to select HOMER3-AS1 silenced cells. To construct HOMER3 stably overexpressed and control HCC cells, HOMER3 overexpression plasmid or empty plasmid pcDNA3.1 was transfected into SK-HEP-1 cells. The transfected cells were treated with 800 µg/ml neomycin 48 h after transfection for 4 weeks to select HOMER3 overexpressed HCC cells. To construct HOMER3-AS1 overexpressed and concurrently HOMER3 depleted HCC cells, HOMER3-AS1 overexpressed SK-HEP-1 cells were infected with shRNA lentivirus repressing HOMER3. The infected cells were treated with 2 µg/ml puromycin and 800 µg/ml neomycin 96 h after infection for four weeks to select HOMER3-AS1 overexpressed and concurrently HOMER3-AS1 silenced cells.

### Cell proliferation, apoptosis, migration, and invasion assays

Cell Counting Kit-8 (CCK-8) and 5-ethynyl-2’-deoxyuridine (EdU) incorporation experiments were undertaken to measure cell proliferation. For CCK-8 experiments, 2000 indicated HCC cells re-suspended in 100 µL media were plated onto a 96-well plate. After culturing for 4d, 10 µL CCK-8 reagents (Dojindo, Kumamoto, Japan) were added to each well. After culture for another 2 h, a microplate reader (BioTek, Winooski, VT, USA) was used to measure the absorbance at 450 nm to indicate the number of viable cells. For EdU incorporation experiments, indicated HCC cells were treated with 50 μM EdU (RiboBio, Guangzhou, China) for 2 h. After being fixed in 4% paraformaldehyde for 30 min and permeabilized using 0.5% TritonX-100 for 10 min, the cells were stained with Apollo dye solution (RiboBio). The cell nucleus was further stained using DAPI. The number of EdU-positive and proliferative cells was detected using a fluorescence microscope (Carl Zeiss, Oberkochen, Germany). Cell apoptosis was detected using the Caspase-3 Activity Assay Kit (Cell Signaling Technology, Danvers, MA, USA) strictly following the provided protocol. Cell migration and invasion were evaluated by transwell migration and invasion assays as we previously described [23].

### Co-culture assay

PMA-stimulated THP-1 cells were added into the upper chamber of 8 μm pore transwell inserts (Corning, NY, USA). SK-HEP-1 cells with HOMER3-AS1 overexpression or silencing were plated into the lower chamber. After culture for 48 h, THP-1 cells remaining in the upper chamber were removed. The migrated THP-1 cells were fixed, stained, and detected using a microscope. SK-HEP-1 cells with HOMER3-AS1 overexpression or silencing were plated into the upper chamber of 0.4 μm pore transwell inserts (Corning). PMA-stimulated THP-1 cells were plated into the lower chamber. After co-culture for 96 h, THP-1 cells were collected to extract RNA. Genes expression in THP-1 cells were detected by qRT-PCR. THP-1 cellular proliferation was detected by CCK-8 experiments.

### Subcutaneous and orthotopic models

Five-week-old male BALB/C athymic nude mice were purchased from Shanghai SLAC Laboratory Animal Co. and fed in Specific Pathogen Free (SPF) conditions. Luciferase-labelled SK-HEP-1 cells with HOMER3-AS1 overexpression or silencing were subcutaneously injected into nude mice. When the subcutaneous xenografts grew to about 0.7 cm in diameter, they were removed and cut into small pieces, which were then transplanted into the liver of nude mice. At the 14^th^ day after transplantation, the tumors were detected by bioluminescence imaging using IVIS^@^ Lumina II system (Caliper Life Sciences, Hopkinton, MA, USA). No statistical method was used to determine the sample size. The experiments were not randomized. The investigators performed the bioluminescence imaging were blinded to mouse allocation. The animal experiments were undertaken with the approval of the Animal Ethics Committee of Affiliated Hospital of Youjiang Medical University for Nationalities.

### Immunohistochemistry (IHC) and Immunofluorescence (IF)

The liver orthotopic xenografts were used to perform IHC staining as previously described with primary antibodies against Ki67 (#9027, 1:400, Cell Signaling Technology) or cleaved caspase-3 (#9664, 1:1000, Cell Signaling Technology) [37]. Human HCC tissues were used to perform IHC staining with primary antibodies against CD163 (#93498, 1:500, Cell Signaling Technology) or HOMER3 (16624-1-AP, 1:200, Proteintech, Chicago, IN, USA). The liver orthotopic xenografts were used to perform IF as previously described with primary antibodies against F4/80 (#30325, 1:400, Cell Signaling Technology) [35]. SK-HEP-1 cells with HOMER3-AS1 overexpression or silencing were used to perform IF as previously described with primary antibodies against β-catenin (#8480, 1:100, Cell Signaling Technology) [25].

### Western blot of total protein and nuclear protein

Total protein was extracted from SK-HEP-1 cells with HOMER3-AS1 stable overexpression or silencing using the RIPA Lysis Buffer (Beyotime, Shanghai, China) added with proteinase inhibitor PMSF (Beyotime). Nuclear protein was extracted from SK-HEP-1 cells with HOMER3-AS1 stable overexpression or silencing using the Nuclear and Cytoplasmic Protein Extraction Kit (Beyotime, Shanghai, China). The extracted total protein and nuclear protein was separated by sodium dodecyl sulfate-polyacrylamide gel electrophoresis and then transferred onto polyvinylidene fluoride membrane. After being blocked with 5% skimmed milk, the membranes were incubated with primary antibodies against HOMER3 (ab97438, 1:1000, Abcam, Cambridge, MA, USA), GAPDH (ab8245, 1:10000, Abcam), β-catenin (#8480, 1:1000, Cell Signaling Technology), or histone H3 (ab1791, 1:1000). After three washes, the membranes were incubated with IRDye 800CW Goat anti-Rabbit IgG (Li-Cor, Lincoln, NE, USA) or IRDye 680RD Goat anti-Mouse IgG (Li-Cor), followed by being scanned on an Odyssey infrared scanner (Li-Cor). GAPDH and histone H3 were used as loading control for total protein and nuclear protein, respectively.

### RNA fluorescence in situ hybridization (FISH)

For in situ detection of HOMER3-AS1 in HCC tissues, the HOMER3-AS1 probes were designed and synthesized by Advanced Cell Diagnostics (Hayward, CA, USA). RNAscope Fluorescent Multiplex Detection Kit (Advanced Cell Diagnostics) was used to perform the hybridization and fluorescence detection.

### Dual-luciferase reporter assays

Wild-type Wnt/β-catenin reporter TOPFlash (Addgene, Watertown, MA, USA) and pRL-TK (Promega, Madison, WI, USA) were co-transfected into SK-HEP-1 cells with HOMER3-AS1 stable overexpression or silencing. pRL-TK encodes Renilla luciferase and was used as endogenous control. After culture for another 48 h, the Firefly luciferase and Renilla luciferase activities were measured using the Dual-Luciferase Reporter Assay System (Promega). The results were calculated as the ratio of Firefly luciferase activity to Renilla luciferase activity.

### Statistical analysis

All statistical analyses were conducted using the GraphPad Prism 6.0 Software. The detailed statistical methods were shown in the figure and table legends. *p* < 0.05 was considered statistically significant.

## Results

### HOMER3-AS1 is correlated with advanced stage and poor prognosis in HCC

**Fig. 1:**
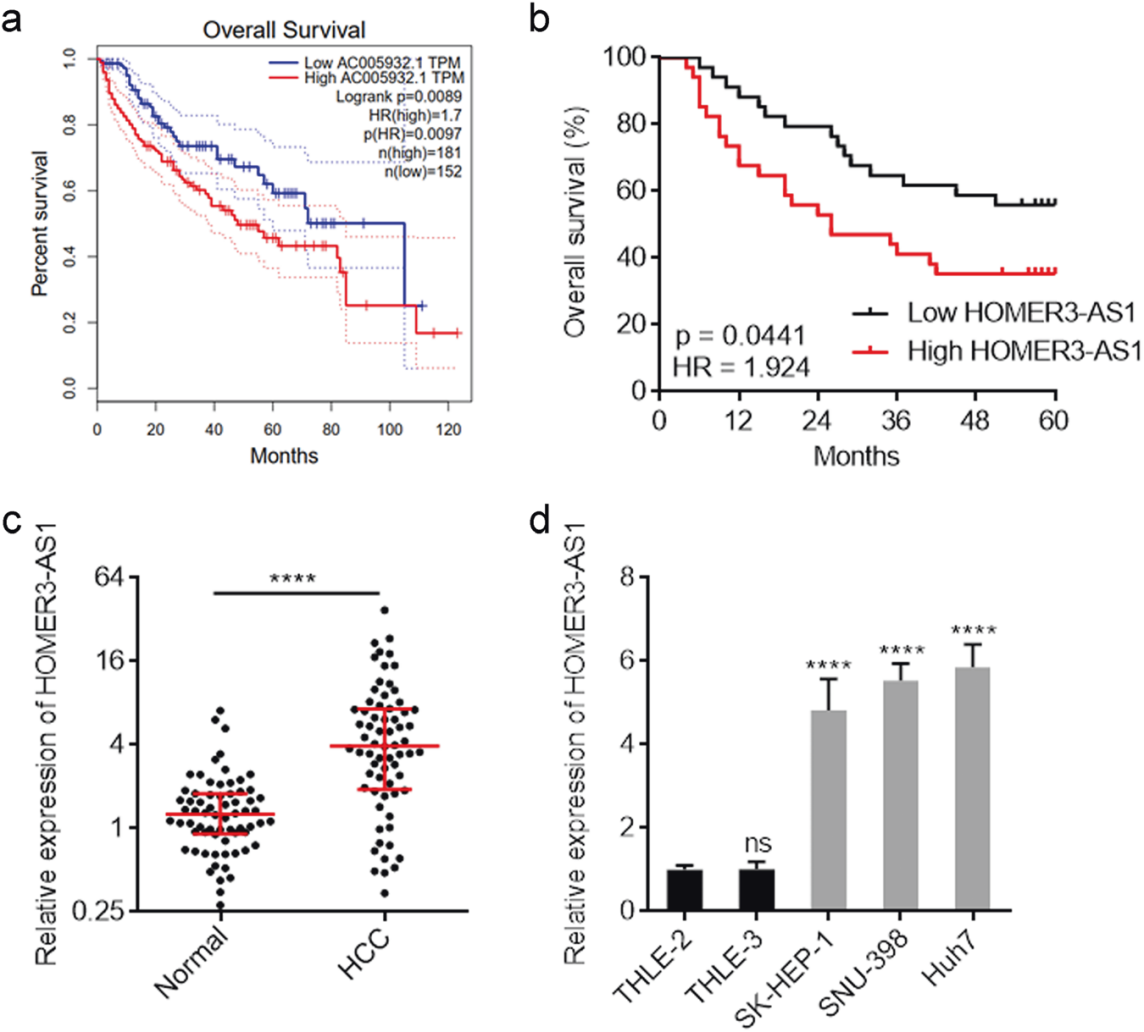
The expression and clinical relevance of HOMER3-AS1 in HCC. **a** The correlation between HOMER3-AS1 (AC005932.1) expression and overall survival according to TCGA LIHC dataset, analyzed by GEPIA. **b** Kaplan-Meier survival analysis of the correlation between HOMER3-AS1 expression and overall survival in our HCC cohort. *n* = 68, *p* = 0.0441, HR = 1.924 by log-rank test. **c** HOMER3-AS1 expression in 68 pairs of HCC tissues and adjacent noncancerous liver tissues was measured by qRT-PCR. ****p < 0.0001 by Wilcoxon matched-pairs signed-rank test. **d** HOMER3-AS1 expression in immortalized human liver cell lines THLE-2 and THLE-3, and human HCC cell lines SK-HEP-1, SNU-398, and Huh7 were measured by qRT-PCR. Results are presented as mean ± SD based on three independent experiments. ****p < 0.0001, ns, not significant, by one-way ANOVA followed by Dunnett’s multiple comparisons test.

The correlation between HOMER3-AS1 expression levels and overall survival in HCC was analyzed by Gene Expression Profiling Interactive Analysis (GEPIA) (http://gepia.cancer-pku.cn/index.html) based on the cancer genome atlas (TCGA) liver hepatocellular carcinoma (LIHC) dataset [38]. The results revealed that high HOMER3-AS1 (AC005932.1) expression was correlated with poor overall survival (Fig. 1a). To further test the correlation between HOMER3-AS1 expression levels and prognosis in HCC, we collected 68 HCC tissues and measured HOMER3-AS1 expression by qRT-PCR. Consistently, high HOMER3-AS1 expression was correlated with poor overall survival in our HCC cohort (Fig. 1b). Analyses of the correlations between HOMER3-AS1 expression levels and clinicopathological characteristics revealed that high HOMER3-AS1 expression was correlated with advanced BCLC stage, high serum AFP concentration, and microvascular invasion (Table 1). In these 68 HCC cases, the expression of HOMER3-AS1 in paired adjacent noncancerous liver tissues was also measured. The results revealed that HOMER3-AS1 was significantly upregulated in HCC tissues compared with noncancerous liver tissues (Fig. 1c). Furthermore, the expression of HOMER3-AS1 in immortalized human liver cell lines THLE-2 and THLE-3, and human HCC cell lines SK-HEP-1, SNU-398, and Huh7 was measured. The result revealed that HOMER3-AS1 was also significantly upregulated in HCC cell lines compared with immortalized liver cell lines (Fig. 1d). The high expression and clinical relevance of HOMER3-AS1 in HCC implied that HOMER3-AS1 might be an oncogenic lncRNA in HCC.

### HOMER3-AS1 promotes the malignant phenotype of HCC cells

**Fig. 2:**
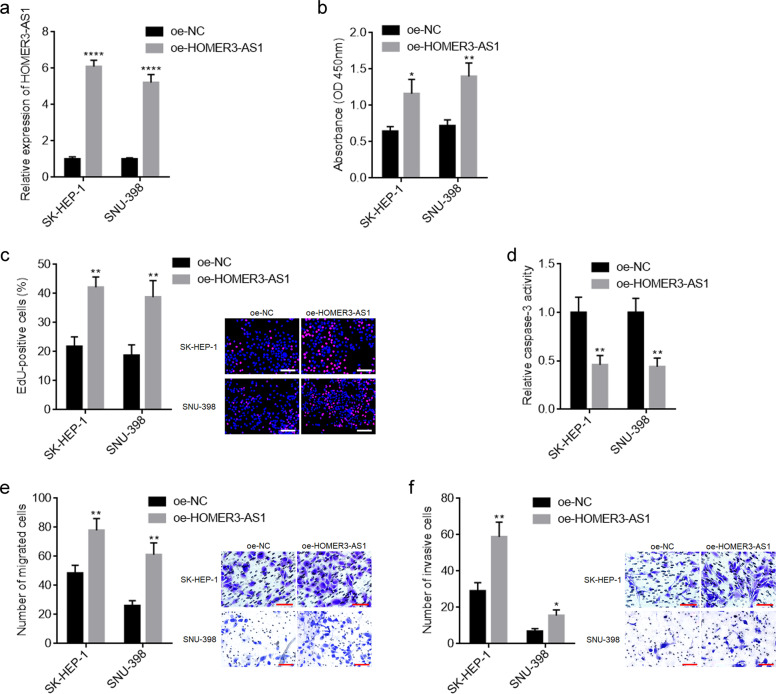
Overexpression of HOMER3-AS1 promoted HCC cellular malignant phenotype. **a** HOMER3-AS1 expression in SK-HEP-1 and SNU-398 cells with HOMER3-AS1 stable overexpression was measured by qRT-PCR. **b** Cell proliferation of SK-HEP-1 and SNU-398 cells with HOMER3-AS1 stable overexpression was determined by CCK-8 experiments. **c** Cell proliferation of SK-HEP-1 and SNU-398 cells with HOMER3-AS1 stable overexpression was determined by EdU incorporation experiments. Scale bars, 100 µm. The red color indicates EdU-positive nucleuses and blue color indicates all cellular nucleuses. Results are shown as the ratio of EdU-positive nucleuses to all cellular nucleuses. **d** Cell apoptosis of SK-HEP-1 and SNU-398 cells with HOMER3-AS1 stable overexpression was determined by caspase-3 activity assays. **e** Cell migration of SK-HEP-1 and SNU-398 cells with HOMER3-AS1 stable overexpression was determined by transwell migration assays. Scale bars, 100 µm. **f** Cell invasion of SK-HEP-1 and SNU-398 cells with HOMER3-AS1 stable overexpression was determined by transwell invasion assays. Scale bars, 100 µm. Results are presented as mean ± SD based on three independent experiments. *p < 0.05, **p < 0.01, ****p < 0.0001 by Student’s *t*-test.

To explore the potential roles of HOMER3-AS1 in HCC, HOMER3-AS1 was stably overexpressed in SK-HEP-1 and SNU-398 cells via transfection of HOMER3-AS1 overexpression plasmid (Fig. 2a). CCK-8 and EdU incorporation experiments revealed that overexpression of HOMER3-AS1 promoted HCC cellular proliferation (Fig. 2b, c). Caspase-3 activity assays revealed that overexpression of HOMER3-AS1 reduced HCC cellular apoptosis (Fig. 2d). Transwell migration and invasion experiments revealed that overexpression of HOMER3-AS1 promoted HCC cellular migration and invasion (Fig. 2e, f). Furthermore, HOMER3-AS1 was stably overexpressed in immortalized liver cell line THLE-2 (Supplementary Fig. 1a). CCK-8 and EdU incorporation experiments revealed that overexpression of HOMER3-AS1 also promoted THLE-2 cellular proliferation (Supplementary Fig. 1b, c). Caspase-3 activity assays revealed that overexpression of HOMER3-AS1 also reduced THLE-2 cellular apoptosis (Supplementary Fig. 1d). Transwell migration and invasion experiments revealed that overexpression of HOMER3-AS1 also promoted HCC cellular migration and invasion (Supplementary Fig. 1e, f). These data suggested that overexpression of HOMER3-AS1 exerts oncogenic roles in HCC.

HOMER3-AS1 was stably silenced in SK-HEP-1 and Huh7 cells via infection of two independent HOMER3-AS1 specific shRNA lentiviruses (Supplementary Fig. 2a). CCK-8 and EdU incorporation experiments revealed that HOMER3-AS1 silencing restricted HCC cellular proliferation (Supplementary Fig. 2b, c). Caspase-3 activity assays revealed that HOMER3-AS1 silencing promoted HCC cellular apoptosis (Supplementary Fig. 2d). Transwell migration and invasion experiments revealed that HOMER3-AS1 silencing restricted HCC cellular migration and invasion (Supplementary Fig. 2e, f). These data further support the oncogenic roles of HOMER3-AS1 in HCC.

### HOMER3-AS1 promoted HCC tumor progression in vivo

**Fig. 3:**
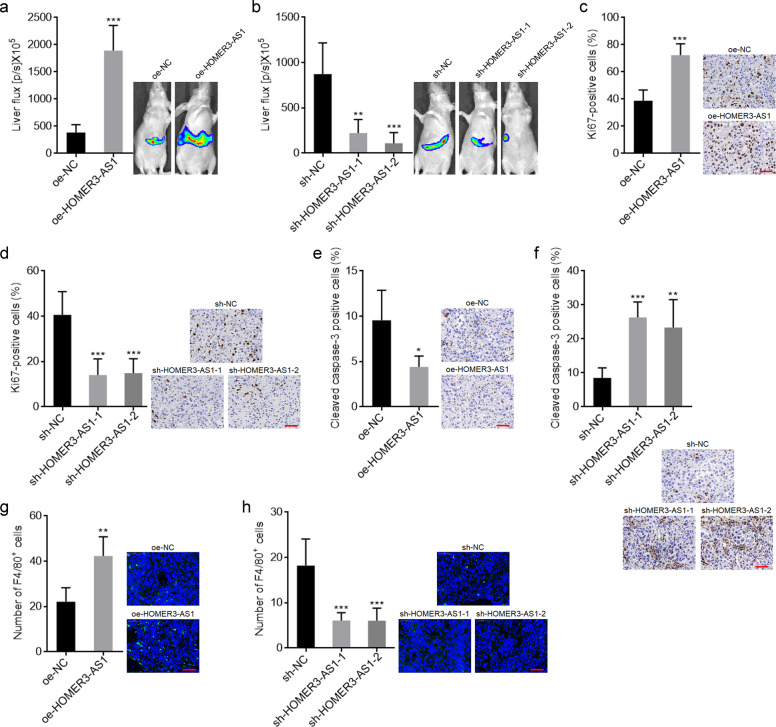
HOMER3-AS1 promoted HCC tumor progression in vivo. **a** Bioluminescence imaging of liver tumors in mice at day 14 after inoculation with small pieces of subcutaneous tumors formed by luciferase labelled SK-HEP-1 cells with HOMER3-AS1 stable overexpression or control. **b** Bioluminescence imaging of liver tumors in mice at day 14 after inoculation with small pieces of subcutaneous tumors formed by luciferase labelled SK-HEP-1 cells with HOMER3-AS1 stable silencing or control. **c** Ki67 IHC staining of liver tumors formed by SK-HEP-1 cells with HOMER3-AS1 stable overexpression or control. Scale bars, 50 µm. **d** Ki67 IHC staining of liver tumors formed by SK-HEP-1 cells with HOMER3-AS1 stable silencing or control. Scale bars, 50 µm. **e** Cleaved caspase-3 IHC staining of liver tumors formed by SK-HEP-1 cells with HOMER3-AS1 stable overexpression or control. Scale bars, 50 µm. **f** Cleaved caspase-3 IHC staining of liver tumors formed by SK-HEP-1 cells with HOMER3-AS1 stable silencing or control. Scale bars, 50 µm. **g** F4/80 IF staining of liver tumors formed by SK-HEP-1 cells with HOMER3-AS1 stable overexpression or control. Scale bars, 50 µm. **h** F4/80 IF staining of liver tumors formed by SK-HEP-1 cells with HOMER3-AS1 stable silencing or control. Scale bars, 50 µm. Results are presented as mean ± SD based on *n* = 5 mice in each group. *p < 0.05, **p < 0.01, ***p < 0.001 by Student’s *t*-test (**a**, **c**, **e**, **g**) or one-way ANOVA followed by Dunnett’s multiple comparisons test (**b**, **d**, **f**, **h**).

To further evaluate the roles of HOMER3-AS1 in HCC xenograft models, small pieces of subcutaneous tumors formed by luciferase labelled SK-HEP-1 cells with HOMER3-AS1 stable overexpression or silencing were orthotopically transplanted into the livers of nude mice. Bioluminescence imaging showed that the xenografts derived from HOMER3-AS1 overexpressed SK-HEP-1 cells were remarkably larger than those derived from control SK-HEP-1 cells (Fig. 3a). Conversely, the xenografts derived from HOMER3-AS1 silenced SK-HEP-1 cells were remarkably smaller than those derived from control SK-HEP-1 cells (Fig. 3b). Proliferation marker Ki67 IHC staining of orthotropic xenografts revealed that the xenografts derived from HOMER3-AS1 overexpressed SK-HEP-1 cells had more Ki67 positive cells than those derived from control SK-HEP-1 cells (Fig. 3c). Conversely, the xenografts derived from HOMER3-AS1 silenced SK-HEP-1 cells had less Ki67 positive cells than those derived from control SK-HEP-1 cells (Fig. 3d). Apoptosis marker cleaved caspase-3 IHC staining of orthotropic xenografts revealed that the xenografts derived from HOMER3-AS1 overexpressed SK-HEP-1 cells had less apoptotic cells than those derived from control SK-HEP-1 cells (Fig. 3e). Conversely, the xenografts derived from HOMER3-AS1 silenced SK-HEP-1 cells had more apoptotic cells than those derived from control SK-HEP-1 cells (Fig. 3f). Macrophage marker F4/80 IF staining of orthotropic xenografts revealed that the xenografts derived from HOMER3-AS1 overexpressed SK-HEP-1 cells had more F4/80^+^ macrophages infiltration than those derived from control SK-HEP-1 cells (Fig. 3g). Conversely, the xenografts derived from HOMER3-AS1 silenced SK-HEP-1 cells had less F4/80^+^ macrophages infiltration than those derived from control SK-HEP-1 cells (Fig. 3h). Collectively, these data suggested that HOMER3-AS1 had oncogenic roles in vivo. HOMER3-AS1 not only promoted HCC cellular malignant phenotype, but also modulated tumor microenvironment.

### HOMER3-AS1 upregulates HOMER3 and activates Wnt/β-catenin signaling

**Fig. 4:**
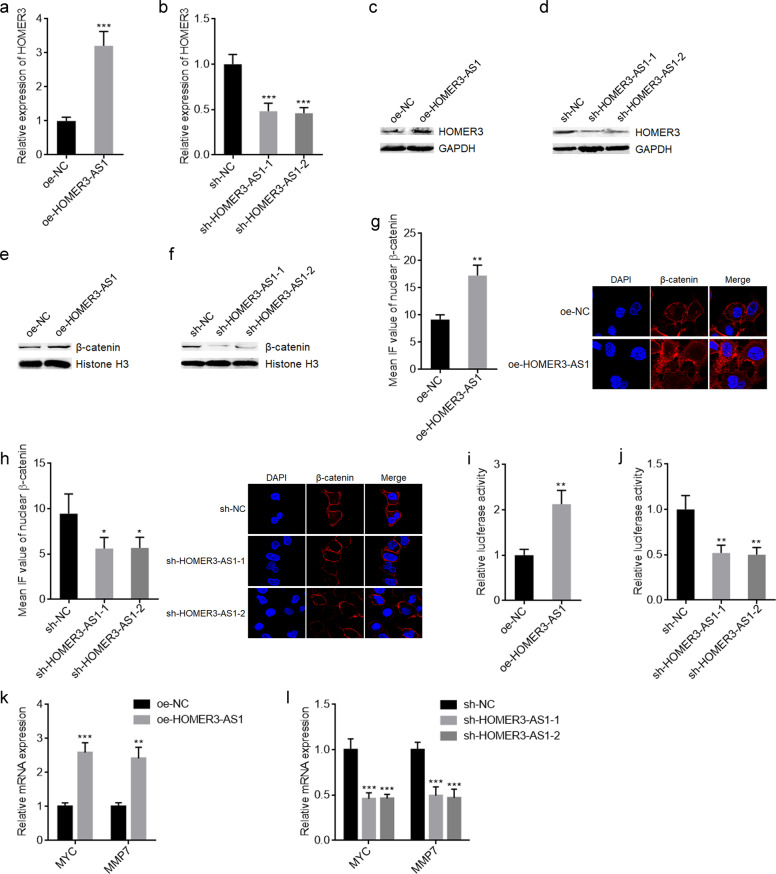
HOMER3-AS1 increased HOMER3 expression and activated Wnt/β-catenin signaling. **a** HOMER3 mRNA levels in SK-HEP-1 cells with HOMER3-AS1 stable overexpression or control was measured by qRT-PCR. **b** HOMER3 mRNA levels in SK-HEP-1 cells with HOMER3-AS1 stable silencing or control were measured by qRT-PCR. **c** HOMER3 protein levels in SK-HEP-1 cells with HOMER3-AS1 stable overexpression or control was measured by western blot. **d** HOMER3 protein levels in SK-HEP-1 cells with HOMER3-AS1 stable silencing or control was measured by qRT-PCR. **e** Nuclear β-catenin protein levels in SK-HEP-1 cells with HOMER3-AS1 stable overexpression or control was measured by western blot. **f** Nuclear β-catenin protein levels in SK-HEP-1 cells with HOMER3-AS1 stable silencing or control was measured by western blot. **g** β-catenin expression in the nucleus of SK-HEP-1 cells with HOMER3-AS1 stable overexpression or control was detected by IF. **h** β-catenin expression in the nucleus of SK-HEP-1 cells with HOMER3-AS1 stable silencing or control was detected by IF. **i** β-catenin reporter TOPFlash and pRL-TK which encodes Renilla luciferase were transfected into SK-HEP-1 cells with HOMER3-AS1 stable overexpression or control. Dual-luciferase reporter assays were undertaken to determine β-catenin transcriptional activity. **j** β-catenin reporter TOPFlash and pRL-TK were transfected into SK-HEP-1 cells with HOMER3-AS1 stable silencing or control. Dual-luciferase reporter assays were undertaken to determine β-catenin transcriptional activity. **k** Wnt/β-catenin downstream targets MYC and MMP7 expressions in SK-HEP-1 cells with HOMER3-AS1 stable overexpression or control. **l** Wnt/β-catenin downstream targets MYC and MMP7 expressions in SK-HEP-1 cells with HOMER3-AS1 stable silencing or control. Results are presented as mean ± SD based on three independent experiments. *p < 0.05, **p < 0.01, ***p < 0.001 by Student’s *t*-test (**a**, **g**, **i**, **k**) or one-way ANOVA followed by Dunnett’s multiple comparisons test (**b**, **h**, **j**, **l**).

HOMER3-AS1 is transcribed in the anti-sense direction of HOMER3. Several antisense strand lncRNAs were revealed to regulate the expressions of their sense strand mRNAs [22, 37]. Thus, we further investigated the potential effects of HOMER3-AS1 on HOMER3. qRT-PCR results revealed that HOMER3 mRNA levels were significantly upregulated after HOMER3-AS1 overexpression and significantly reduced after HOMER3-AS1 silencing (Fig. 4a, b). Western blot results revealed that HOMER3 protein levels were significantly upregulated after HOMER3-AS1 overexpression and significantly reduced after HOMER3-AS1 silencing (Fig. 4c, d). HOMER3 belongs to the HOMER family of density scaffolding proteins and was revealed to promote breast cancer progression via activating Wnt/β-catenin signaling [39]. Thus, we further explored the potential effects of HOMER3-AS1 on Wnt/β-catenin signaling in HCC. Subcellular fractionation and western blot experiments revealed that overexpression of HOMER3-AS1 increased nuclear β-catenin levels and while HOMER3-AS1 silencing reduced nuclear β-catenin levels (Fig. 4e, f). β-catenin IF staining also revealed that nuclear β-catenin increased after HOMER3-AS1 overexpression and reduced after HOMER3-AS1 silencing (Fig. 4g, h). Wnt/β-catenin reporter TOPFlash luciferase activity assays revealed that overexpression of HOMER3-AS1 increased TOPFlash luciferase activity and while HOMER3-AS1 silencing reduced TOPFlash luciferase activity (Fig. 4i, j). Furthermore, Wnt/β-catenin downstream targets MYC and MMP7 were upregulated after HOMER3-AS1 overexpression and downregulated after HOMER3-AS1 silencing (Fig. 4k, l). Collectively, these data suggested that HOMER3-AS1 upregulates HOMER3 and activates Wnt/β-catenin signaling in HCC.

### HOMER3 expression is positively correlated with HOMER3-AS1 expression and poor prognosis in HCC

HOMER3 expression in the same 68 pairs of HCC tissues and adjacent noncancerous liver tissues were measured. Similar with HOMER3-AS1, HOMER3 was significantly upregulated in HCC tissues compared with noncancerous liver tissues (Supplementary Fig. 3a). The expression of HOMER3 was significantly positively correlated with that of HOMER3-AS1 in these 68 HCC tissues (Supplementary Fig. 3b). Similar with HOMER3-AS1, high HOMER3 expression was correlated with poor overall survival in this HCC cohort (Supplementary Fig. 3c). The remarkably positive correlation between HOMER3 and HOMER3-AS1 expression in HCC tissues was also confirmed in the TCGA LIHC dataset, analyzed by GEPIA (Supplementary Fig. 3d). Analyses of the TCGA LIHC dataset by GEPIA also revealed that high HOMER3 expression was correlated with poor overall survival in HCC (Supplementary Fig. 3e). Furthermore, the expressions of HOMER3-AS1 and HOMER3 in HCC tissues were detected by RNA FISH and IHC respectively. The results revealed that both HOMER3-AS1 and HOMER3 were mainly expressed in HCC cells, but not in stromal cells (Supplementary Fig. 3f), supporting clinical relevancy of HOMER3-AS1 and HOMER3 in HCC.

### HOMER3-AS1 promotes HCC cellular malignant phenotype via regulating HOMER3/Wnt/β-catenin

**Fig. 5:**
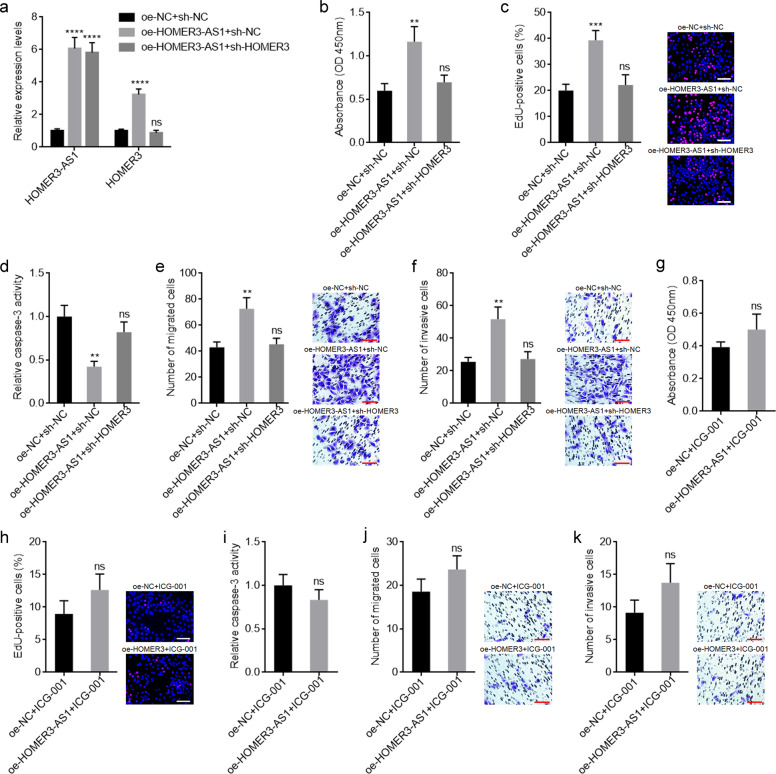
Inhibition of HOMER3/Wnt/β-catenin signaling abolished the oncogenic roles of HOMER3-AS1 in HCC. **a** HOMER3-AS1 and HOMER3 expression in SK-HEP-1 cells with HOMER3-AS1 overexpression and concurrent HOMER3 depletion. **b** Cell proliferation of SK-HEP-1 cells with HOMER3-AS1 overexpression and HOMER3 depletion was determined by CCK-8 experiments. **c** Cell proliferation of SK-HEP-1 cells with HOMER3-AS1 overexpression and HOMER3 depletion was determined by EdU incorporation experiments. Scale bars, 100 µm. **d** Cell apoptosis of SK-HEP-1 cells with HOMER3-AS1 overexpression and HOMER3 depletion was determined by caspase-3 activity assays. **e** Cell migration of SK-HEP-1 cells with HOMER3-AS1 overexpression and HOMER3 depletion was determined by transwell migration assays. Scale bars, 100 µm. **f** Cell invasion of SK-HEP-1 cells with HOMER3-AS1 overexpression and HOMER3 depletion was determined by transwell invasion assays. Scale bars, 100 µm. **g** Cell proliferation of HOMER3-AS1 overexpressed and control SK-HEP-1 cells treated with 10 μM ICG-001 was determined by CCK-8 experiments. **h** Cell proliferation of HOMER3-AS1 overexpressed and control SK-HEP-1 cells treated with 10 μM ICG-001 was determined by EdU incorporation experiments. Scale bars, 100 µm. **i** Cell apoptosis of HOMER3-AS1 overexpressed and control SK-HEP-1 cells treated with 10 μM ICG-001 was determined by caspase-3 activity assays. **j** Cell migration of HOMER3-AS1 overexpressed and control SK-HEP-1 cells treated with 10 μM ICG-001 was determined by transwell migration assays. Scale bars, 100 µm. **k** Cell invasion of HOMER3-AS1 overexpressed and control SK-HEP-1 cells treated with 10 μM ICG-001 was determined by transwell invasion assays. Scale bars, 100 µm. Results are presented as mean ± SD based on three independent experiments. **p < 0.01, ***p < 0.001, ****p < 0.0001, ns, not significant, by one-way ANOVA followed by Dunnett’s multiple comparisons test (**a**-**f**) or Student’s *t*-test (**g**-**k**).

To explore whether HOMER3/Wnt/β-catenin mediates the oncogenic roles of HOMER3-AS1 in HCC, we first detected the potential roles of HOMER3 in HCC. HOMER3 was stably overexpressed in SK-HEP-1 cells via transfection of the HOMER3 overexpression plasmid (Supplementary Fig. 4a). CCK-8 and EdU incorporation experiments revealed that overexpression of HOMER3 promoted HCC cellular proliferation (Supplementary Fig. 4b, c). Caspase-3 activity assays revealed that overexpression of HOMER3 reduced HCC cellular apoptosis (Supplementary Fig. 4d). Transwell migration and invasion experiments revealed that overexpression of HOMER3 promoted HCC cellular migration and invasion (Supplementary Fig. 4e, f). HOMER3 was stably silenced in HOMER3-AS1 overexpressed SK-HEP-1 cells via infection of HOMER3 specific shRNA lentiviruses (Fig. 5a). CCK-8 and EdU incorporation experiments revealed that depletion of HOMER3 abolished the HOMER3-AS1-induced promotion of proliferation (Fig. 5b, c). Caspase-3 activity assays revealed that depletion of HOMER3 abolished the HOMER3-AS1-induced inhibition of apoptosis (Fig. 5d). Transwell migration and invasion experiments revealed that depletion of HOMER3 abolished the HOMER3-AS1-induced promotion of migration and invasion (Fig. 5e, f). Furthermore, HOMER3-AS1 overexpressed and control SK-HEP-1 cells were treated with Wnt/β-catenin inhibitor ICG-001. The blocking of Wnt/β-catenin signaling abolished the HOMER3-AS1-induced promotion of proliferation, migration, and invasion, and inhibition of apoptosis (Fig. 5g–k). Collectively, these data suggested that HOMER3-AS1 promotes HCC cellular malignant phenotype via regulating HOMER3/Wnt/β-catenin axis.

### HOMER3-AS1 induces infiltration and M2-like polarization of macrophages

**Fig. 6:**
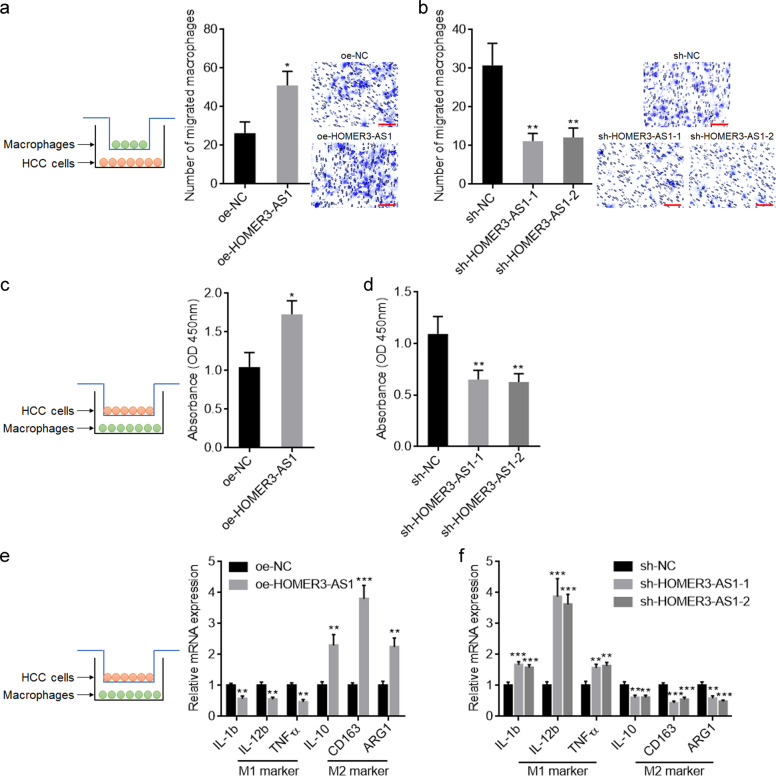
HOMER3-AS1 promoted macrophages infiltration and M2-like polarization. **a** PMA-stimulated THP-1 cells were subjected to migration assays towards SK-HEP-1 cells with HOMER3-AS1 overexpression or control. **b** PMA-stimulated THP-1 cells were subjected to migration assays towards SK-HEP-1 cells with HOMER3-AS1 silencing or control. **c** Cell proliferation of PMA-stimulated THP-1 cells co-cultured with HOMER3-AS1 overexpressed or control SK-HEP-1 cells was determined by CCK-8 experiments. **d** Cell proliferation of PMA-stimulated THP-1 cells co-cultured with HOMER3-AS1 silenced or control SK-HEP-1 cells was determined by CCK-8 experiments. **e** Expression of markers associated with M1 and M2 polarization in PMA-stimulated THP-1 cells co-cultured with HOMER3-AS1 overexpressed or control SK-HEP-1 cells was detected by qRT-PCR. **f** Expression of markers associated with M1 and M2 polarization in PMA-stimulated THP-1 cells co-cultured with HOMER3-AS1 silenced or control SK-HEP-1 cells was detected by qRT-PCR. Results are presented as mean ± SD based on three independent experiments. *p < 0.05, **p < 0.01, ***p < 0.001 by Student’s *t*-test (**a**, **c**, **e**) or one-way ANOVA followed by Dunnett’s multiple comparisons test (**b**, **d**, **f**).

Given that the xenografts formed by HOMER3-AS1 overexpressed HCC cells had more macrophages infiltration, we further explored the potential influences of HOMER3-AS1 on macrophages using in vitro co-culture system by culturing HOMER3-AS1 overexpressed or silenced SK-HEP-1 cells with PMA-treated THP-1 cells (Fig. 6a). HOMER3-AS1 overexpressed SK-HEP-1 cells promoted the migration of macrophages compared with control SK-HEP-1 cells (Fig. 6a). Conversely, HOMER3-AS1 silenced SK-HEP-1 cells inhibited the migration of macrophages compared with control SK-HEP-1 cells (Fig. 6b). At the condition of co-culture, HOMER3-AS1 overexpressed SK-HEP-1 cells enhanced the proliferation of macrophages compared with control SK-HEP-1 cells (Fig. 6c). Conversely, HOMER3-AS1 silenced SK-HEP-1 cells restricted the proliferation of macrophages compared with control SK-HEP-1 cells (Fig. 6d). HOMER3-AS1 overexpressed SK-HEP-1 cells-stimulated macrophages exhibited reduced expression of M1 markers, IL-1b, IL-12b, and TNF-α, with increased expression of M2 markers, IL-10, CD163, and ARG1 (Fig. 6e). Conversely, HOMER3-AS1 silenced SK-HEP-1 cells-stimulated macrophages exhibited increased expression of M1 markers, IL-1b, IL-12b, and TNF-α, with reduced expression of M2 markers, IL-10, CD163, and ARG1 (Fig. 6f). Collectively, these data suggested that HOMER3-AS1 induces macrophages infiltration and M2-like polarization.

### CSF-1 upregulation is responsible for the recruitment and M2-like polarization of macrophages by HOMER3-AS1

To elucidate the mechanisms mediating the roles of HOMER3-AS1 in macrophages recruitment and polarization, we searched the genes with similar expression pattern with HOMER3-AS1 in HCC based on the TCGA LIHC dataset analyzed by GEPIA. CSF-1 ranked the first with a Pearson correlation coefficient of 0.72 (Supplementary Fig. 5a). Similar with HOMER3-AS1, high CSF-1 expression was correlated with poor overall survival in HCC based on the TCGA LIHC dataset analyzed by GEPIA (Supplementary Fig. 5b). CSF-1 expression in the same 68 HCC tissues of our cohort were measured. The expression of CSF-1 was significantly positively correlated with that of HOMER3-AS1 in our HCC cohort (Supplementary Fig. 5c). Similar with HOMER3-AS1, high CSF-1 expression was correlated with poor overall survival in this HCC cohort (Supplementary Fig. 5d). CSF-1 is frequently reported to be a critical regulator of macrophages generation, differentiation, and function [36, 40]. Thus, we further detected the correlation between HOMER3-AS1, CSF-1, and macrophages recruitment in HCC tissues. CD163 IHC staining was performed in these 68 HCC tissues to indicate macrophages infiltration and polarization. The results revealed that the HCC tissues with high levels of CD163^+^ macrophages also had higher HOMER3-AS1 and CSF-1 expression levels than those HCC tissues with low levels of CD163^+^ macrophages (Supplementary Fig. 5e).

**Fig. 7:**
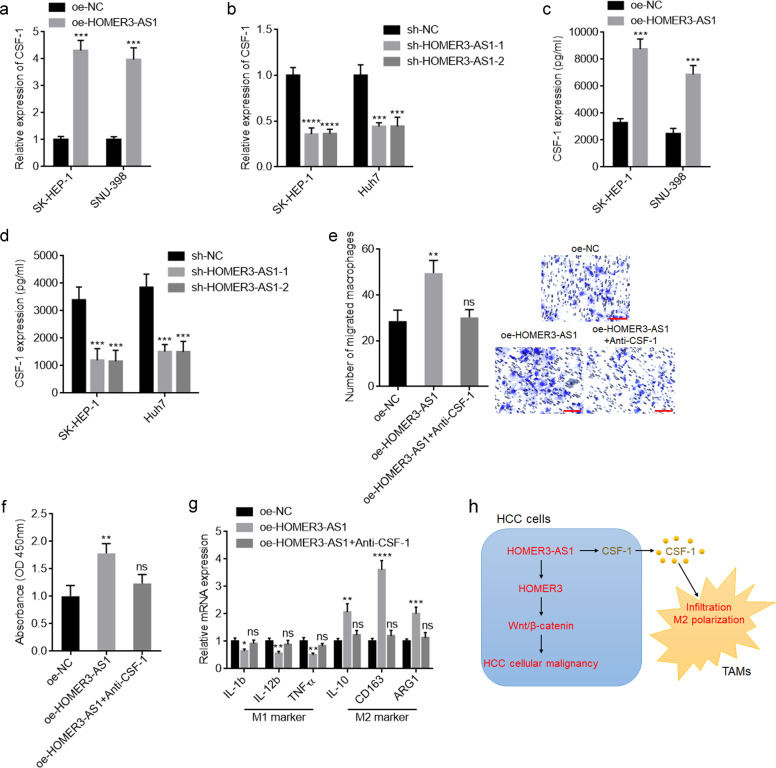
HOMER3-AS1 promoted macrophages infiltration and M2-like polarization via upregulating CSF-1. **a** CSF-1 expression in SK-HEP-1 and SNU-398 cells with HOMER3-AS1 stable overexpression was measured by qRT-PCR. **b** CSF-1 expression in SK-HEP-1 and Huh7 cells with HOMER3-AS1 stable silencing was measured by qRT-PCR. **c** CSF-1 secretion from SK-HEP-1 and SNU-398 cells with HOMER3-AS1 stable overexpression was measured by ELISA. **d** CSF-1 secretion from SK-HEP-1 and Huh7 cells with HOMER3-AS1 stable silencing was measured by ELISA. **e** PMA-stimulated THP-1 cells were subjected to migration assays towards CM from HOMER3-AS1 overexpressed SK-HEP-1 cells treated with or without anti-CSF-1. **f** Cell proliferation of PMA-stimulated THP-1 cells after culture with CM from HOMER3-AS1 overexpressed SK-HEP-1 cells treated with or without anti-CSF-1. **g** Expression of markers associated with M1 and M2 polarization in PMA-stimulated THP-1 cells after culture with CM from HOMER3-AS1 overexpressed SK-HEP-1 cells treated with or without anti-CSF-1. **h** Schematic model of the roles of HOMER3-AS1 in regulating HCC cellular malignancy and TAMs infiltration and polarization. Results are presented as mean ± SD based on three independent experiments. *p < 0.05, **p < 0.01, ***p < 0.001, ****p < 0.0001, ns, not significant, by Student’s *t*-test (**a**, **c**) or one-way ANOVA followed by Dunnett’s multiple comparisons test (**b**, **d**-**g**).

CSF-1 was significantly upregulated in SK-HEP-1 and SNU-398 cells with HOMER3-AS1 overexpression compared with control SK-HEP-1 and SNU-398 cells (Fig. 7a). Conversely, the CSF-1 expression level was significantly reduced after HOMER3-AS1 silencing (Fig. 7b). Furthermore, ELISA results revealed that HCC cells with HOMER3-AS1 overexpression had increased CSF-1 secretion compared with control cells (Fig. 7c). Conversely, HOMER3-AS1 silencing reduced CSF-1 secretion (Fig. 7d). To elucidate whether the induced secretion of CSF-1 by HOMER3-AS1 mediates the roles of HOMER3-AS1 in inducing macrophages infiltration and polarization, conditioned medium (CM) from SK-HEP-1 cells with HOMER3-AS1 overexpression or control was used to stimulate PMA-treated THP-1 cells. Similar with co-culture, CM from HOMER3-AS1 overexpressed SK-HEP-1 cells also promoted macrophages migration, proliferation, and M2 polarization (Fig. 7e–g). Anti-CSF-1 treatment reversed HOMER3-AS1-induced macrophages migration, proliferation, and M2 polarization (Fig. 7e–g). Collectively, these data suggested that HOMER3-AS1 upregulated CSF-1 expression and secretion, which further induced macrophages recruitment and M2-like polarization.

## Discussion

Increasing understandings of the crosstalk between tumor cells and tumor microenvironment (TME) have driven advances in immunotherapy [41–44]. Immune checkpoints inhibitors, such as anti-PD-1 antibody nivolumab and pembrolizumab, have been approved for HCC treatment [45, 46]. However, only about 14% of HCC patients are sensitive to anti-PD-1 antibody. Furthermore, frequent drug resistances to anti-PD-1 antibody were also found in clinical therapy [47]. Thus, further revealing the critical molecules driving HCC progression via mediating the crosstalk between HCC cells and TME would provide novel avenues for HCC therapy.

In this study, we identified a novel HCC-related lncRNA HOMER3-AS1. *HOMER3-AS1* is located in chromosome 19p13.11. HOMER3-AS1 is transcribed in the anti-sense direction of HOMER3 and has 1041 nucleotides in length. Both RNA-sequencing data of the TCGA LIHC project and qRT-PCR detection of our HCC cohort revealed that high expression of HOMER3-AS1 is correlated with poor overall survival of HCC patients. Furthermore, our data also revealed that HOMER3-AS1 is upregulated in HCC tissues and correlated with advanced BCLC stage, high serum AFP concentration, and microvascular invasion. Therefore, these findings suggested HOMER3-AS1 as a potential prognostic biomarker for HCC. The positive correlation between HOMER3-AS1 and AFP suggested that HOMER3-AS1 may be a diagnostic biomarker for HCC, which needs further investigation.

Gain- and loss-of functions experiments revealed that HOMER3-AS1 has oncogenic roles in HCC. HOMER3-AS1 drives HCC progression in vitro and in vivo. HOMER3-AS1 enhances HCC cellular malignant phenotypes, including the promotion of HCC cellular proliferation, migration, and invasion, and the inhibition of HCC cellular apoptosis. Moreover, HOMER3-AS1 was also found to facilitate macrophages infiltration and M2-like polarization. Therefore, HOMER3-AS1 drives HCC progression not only by regulating the behaviors of HCC cells, but also by educating macrophages. Our findings also showed that depletion of HOMER3-AS1 significantly represses HCC progression in vitro and in vivo through reducing HCC cellular malignant phenotypes and macrophages recruitment and M2 polarization. HOMER3-AS1 represents a potential therapeutic target for HCC.

As a class of regulatory non-coding RNAs, lncRNAs exert their oncogenic or tumor-suppressive roles mainly through modulating critical oncogenic or tumor suppressive proteins and signaling pathways [24, 48, 49]. lncRNA PHAROH promotes HCC cellular proliferation and migration via upregulating MYC protein expression [48]. LncRNA MRCCAT1 promotes clear cell renal cell carcinoma metastasis through activating MAPK signaling [49]. lncRNA CASC9 enhances HCC cellular viability via activating AKT signaling [24]. Apart from MAPK and AKT signaling, other signaling pathways were also revealed to be involved in HCC, such as Wnt/β-catenin, TGF-β, NF-κB, and Hedgehog signaling pathways [50–53]. Here, we found that HOMER3-AS1 activated Wnt/β-catenin signaling via upregulating HOMER3. Wnt/β-catenin signaling is frequently reported to be involved in stemness and malignant progression of HCC [54]. In this study, we also found that blocking of Wnt/β-catenin signaling abolished the oncogenic roles of HOMER3-AS1 in HCC cellular malignant phenotypes. Anti-sense lncRNAs could modulate the expression of their sense strand genes at transcriptional, post-transcriptional, or translational levels [22, 37]. GPC3-AS1 epigenetically activates *GPC3* transcription [22]. PXN-AS1-L enhances PXN mRNA stability, and while PXN-AS1-S represses PXN mRNA translation [37]. Here, we found that HOMER3-AS1 upregulates HOMER3 mRNA and protein levels. Thus, HOMER3-AS1 may transcriptionally activate *HOMER3* expression or increase HOMER3 mRNA stability, which need further investigation.

Except for the roles of HOMER3-AS1 in enhancing HCC cellular malignant phenotypes, HOMER3-AS1 was also found to induce macrophages recruitment and M2-like polarization. Several molecules were reported to mediate the crosstalk between cancer cells and macrophages [35, 36, 55]. Tumor cells secrete sonic hedgehog to drive TAMs M2 polarization [55]. OPN derived from HCC cells facilitate macrophages migration and M2 polarization through activating the CSF-1/CSF1R pathway [35]. CircASAP1 promotes macrophage infiltration via modulating the miR-326/miR-532-5p-CSF-1 pathway [36]. Combining bioinformatics analyses and experimental verification, we identified CSF-1 as the critical downstream target of HOMER3-AS1 and the mediator of the roles of HOMER3-AS1 in inducing macrophages recruitment and M2 polarization. The expression of CSF-1 is significantly positively correlated with that of HOMER3-AS1 in HCC tissues, verified in the TCGA LIHC dataset and our HCC cohort. The expression levels of CSF-1 and HOMER3-AS1 are both positively correlated with the number of CD163 positive macrophages. These clinical data supported the HOMER3-AS1/CSF-1/macrophages regulatory axis. The detailed molecular mechanisms of how HOMER3-AS1 regulates CSF-1 need further investigation.

In summary, we identified lncRNA HOMER3-AS1 as a novel HCC-related lncRNA, which is highly expressed in HCC tissues and correlated with poor survival of HCC patients. HOMER3-AS1 enhances HCC cellular malignant phenotypes via upregulating HOMER3 and activating Wnt/β-catenin signaling. HOMER3-AS1 facilitates macrophages infiltration and M2-like polarization via upregulating CSF-1 and inducing CSF-1 secretion (Fig. 7h). These findings suggested HOMER3-AS1 as a potential prognostic biomarker and therapeutic target for HCC.

## Supplementary information

Supplementary Figure Legends

Supplementary Figure 1

Supplementary Figure 2

Supplementary Figure 3

Supplementary Figure 4

Supplementary Figure 5

## Data Availability

The datasets generated and/or analyzed during the current study are available from the corresponding author on reasonable request.
