# Supplementary material for: Long non-coding RNA HOMER3-AS1 drives hepatocellular carcinoma progression via modulating the behaviors of both tumor cells and macrophages
Source: Cell Death Dis. 2021 Nov 23;12(12):1103. doi: 10.1038/s41419-021-04309-z (PMC8611033; doi:10.1038/s41419-021-04309-z)
Supplement: Supplementary file 1 — Supplementary Figure Legends [file 41419_2021_4309_MOESM1_ESM.docx]

**Supplementary Figure legends**

**Supplementary Fig. 1 Overexpression of HOMER3**-**AS1 promoted THLE-2 cellular proliferation, migration, and invasion, and reduced THLE-2 cellular apoptosis.** **a** HOMER3-AS1 expression in THLE-2 cells with HOMER3-AS1 stable overexpression was measured by qRT-PCR. **b** Cell proliferation of THLE-2 cells with HOMER3-AS1 stable overexpression was determined by CCK-8 experiments. **c** Cell proliferation of THLE-2 cells with HOMER3-AS1 stable overexpression was determined by EdU incorporation experiments. Scale bars, 100 µm. Red color indicates EdU-positive nucleuses and blue color indicates all cellular nucleuses. Results are shown as the ratio of EdU-positive nucleuses to all cellular nucleuses. **d** Cell apoptosis of THLE-2 cells with HOMER3-AS1 stable overexpression was determined by caspase-3 activity assays. **e** Cell migration of THLE-2 cells with HOMER3-AS1 stable overexpression was determined by transwell migration assays. Scale bars, 100 µm. **f** Cell invasion of THLE-2 cells with HOMER3-AS1 stable overexpression was determined by transwell invasion assays. Scale bars, 100 µm. Results are presented as mean ± SD based on three independent experiments. **p < 0.01, ***p < 0.001 by Student’s *t*-test.

**Supplementary Fig. 2 HOMER3-AS1 silencing restricted HCC cellular malignant phenotype.** **a** HOMER3-AS1 expression in SK-HEP-1 and Huh7 cells with HOMER3-AS1 stable silencing was measured by qRT-PCR. **b** Cell proliferation of SK-HEP-1 and Huh7 cells with HOMER3-AS1 stable silencing was determined by CCK-8 experiments. **c** Cell proliferation of SK-HEP-1 and Huh7 cells with HOMER3-AS1 stable silencing was determined by EdU incorporation experiments. Scale bars, 100 µm. **d** Cell apoptosis of SK-HEP-1 and Huh7 cells with HOMER3-AS1 stable silencing was determined by caspase-3 activity assays. **e** Cell migration of SK-HEP-1 and Huh7 cells with HOMER3-AS1 stable silencing was determined by transwell migration assays. Scale bars, 100 µm. **f** Cell invasion of SK-HEP-1 and Huh7 cells with HOMER3-AS1 stable silencing was determined by transwell invasion assays. Scale bars, 100 µm. Results are presented as mean ± SD based on three independent experiments. **p < 0.01, ***p < 0.001, ****p < 0.0001 by one-way ANOVA followed by Dunnett's multiple comparisons test.

**Supplementary Fig. 3** **HOMER3 expression is positively correlated with HOMER3-AS1 expression and poor prognosis in HCC.** **a** HOMER3 expression in 68 pairs of HCC tissues and adjacent noncancerous liver tissues was measured by qRT-PCR. ****p < 0.0001 by Wilcoxon matched-pairs signed rank test. **b** The correlation between HOMER3 and HOMER3-AS1 expression in these 68 HCC tissues. p < 0.0001, R = 0.6198 by Spearman correlation analysis. **c** Kaplan-Meier survival analysis of the correlation between HOMER3 expression and overall survival in these 68 HCC cases. p = 0.0448, HR = 1.920 by log-rank test. **d** The correlation between HOMER3 and HOMER3-AS1 (AC005932.1) expression in HCC tissues according to TCGA LIHC dataset, analyzed by GEPIA. **e** The correlation between HOMER3 expression and overall survival according to TCGA LIHC dataset, analyzed by GEPIA. **f** RNA FISH was performed to detect the expression and location of HOMER3-AS1 in HCC tissues. IHC staining was performed to detect the expression and location of HOMER3 in HCC tissues.

**Supplementary Fig. 4** **Overexpression of HOMER3 promoted HCC cellular malignant phenotype.** **a** HOMER3 expression in SK-HEP-1 cells with HOMER3 stable overexpression was measured by qRT-PCR. **b** Cell proliferation of SK-HEP-1 cells with HOMER3 stable overexpression was determined by CCK-8 experiments. **c** Cell proliferation of SK-HEP-1 cells with HOMER3 stable overexpression was determined by EdU incorporation experiments. Scale bars, 100 µm. **d** Cell apoptosis of SK-HEP-1 cells with HOMER3 stable overexpression was determined by caspase-3 activity assays. **e** Cell migration of SK-HEP-1 cells with HOMER3 stable overexpression was determined by transwell migration assays. Scale bars, 100 µm. **f** Cell invasion of SK-HEP-1 cells with HOMER3 stable overexpression was determined by transwell invasion assays. Scale bars, 100 µm. Results are presented as mean ± SD based on three independent experiments. *p < 0.05, **p < 0.01, ***p < 0.001 by Student’s *t*-test.

**Supplementary Fig. 5** **CSF-1 expression is positively correlated with HOMER3-AS1 expression, macrophages infiltration, and poor prognosis in HCC.** **a** The correlation between CSF-1 and HOMER3-AS1 (AC005932.1) expression in HCC tissues according to TCGA LIHC dataset, analyzed by GEPIA. **b** The correlation between CSF-1 expression and overall survival according to TCGA LIHC dataset, analyzed by GEPIA. **c** The correlation between CSF-1 and HOMER3-AS1 expression in our HCC cohort. n = 68, p < 0.0001, R = 0.7164 by Spearman correlation analysis. **d** Kaplan-Meier survival analysis of the correlation between CSF-1 expression and overall survival in our HCC cohort. n = 68, p = 0.0349, HR = 1.983 by log-rank test. **e** HOMER3-AS1 and CSF-1 expression levels in HCC tissues with high or low levels of CD163^+^ macrophages. Scale bars, 50 µm. The median CD163 staining intensity was used as the cutoff. n = 68, ***p < 0.001, ****p < 0.0001 by Mann Whitney test.
