# Supplementary figures and images for: Long non-coding RNA HOMER3-AS1 drives hepatocellular carcinoma progression via modulating the behaviors of both tumor cells and macrophages
Source: Cell Death Dis. 2021 Nov 23;12(12):1103. doi: 10.1038/s41419-021-04309-z (PMC8611033; doi:10.1038/s41419-021-04309-z)

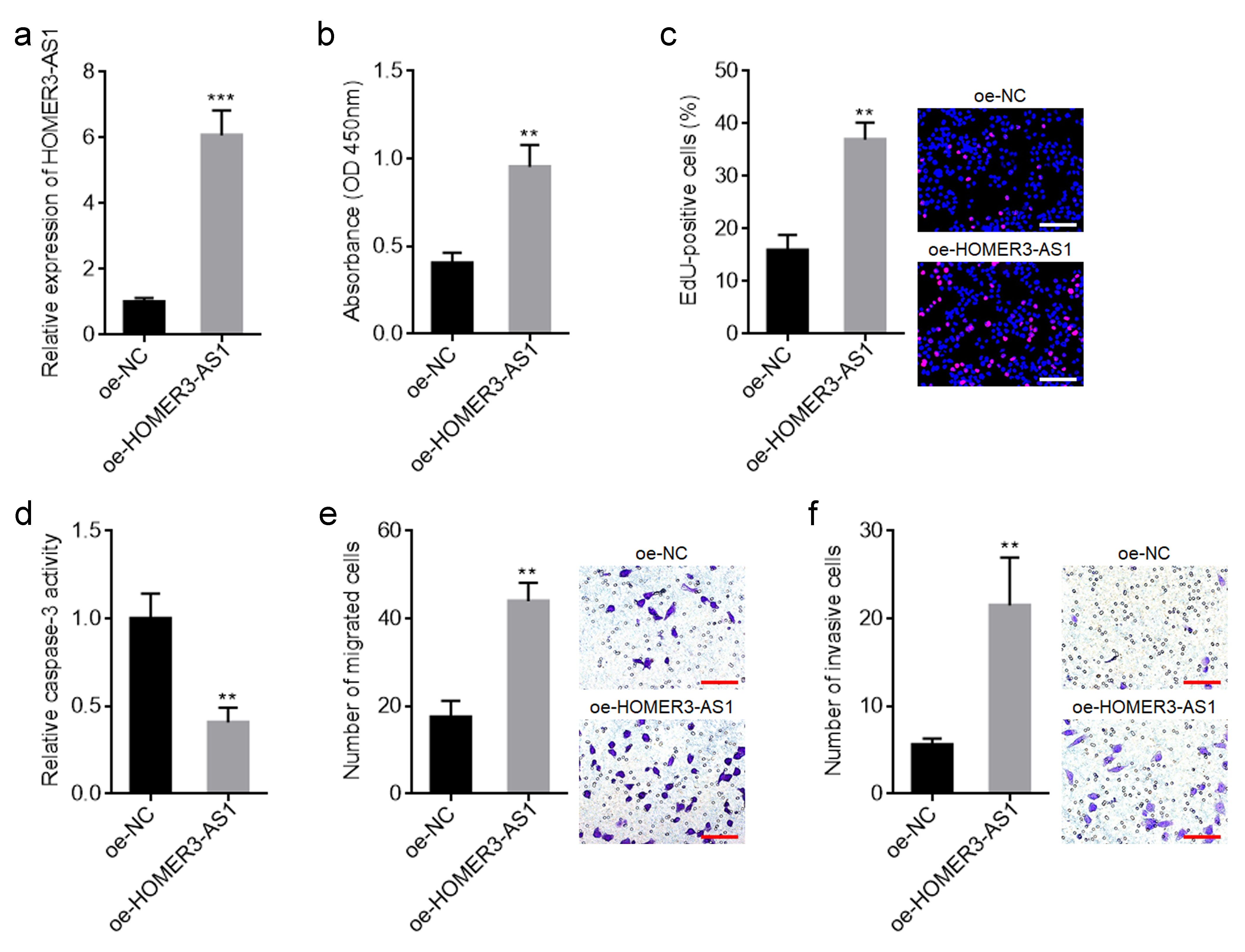

Supplement: Supplementary file 2 — Supplementary Figure 1 [file 41419_2021_4309_MOESM2_ESM.tif]

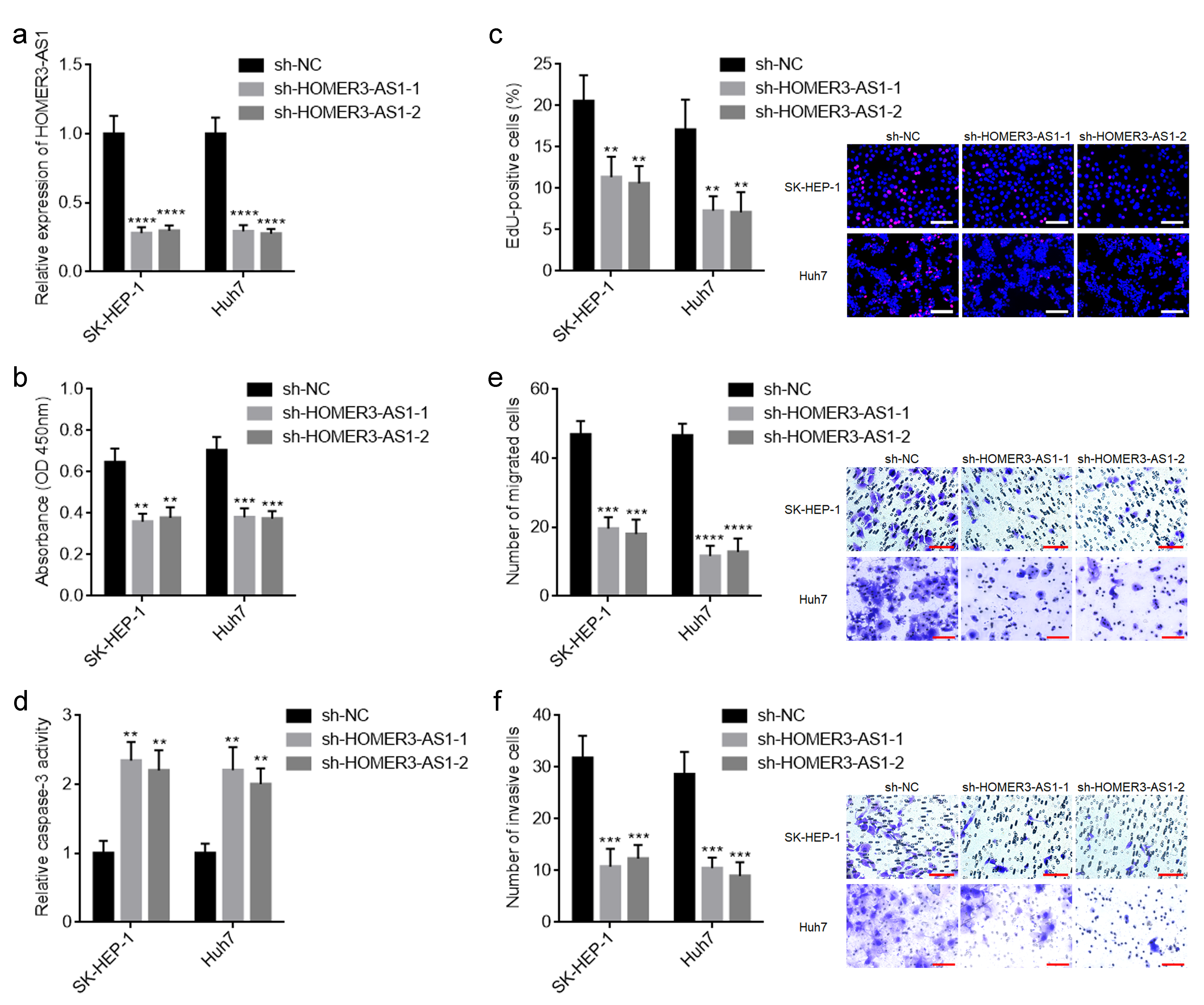

Supplement: Supplementary file 3 — Supplementary Figure 2 [file 41419_2021_4309_MOESM3_ESM.tif]

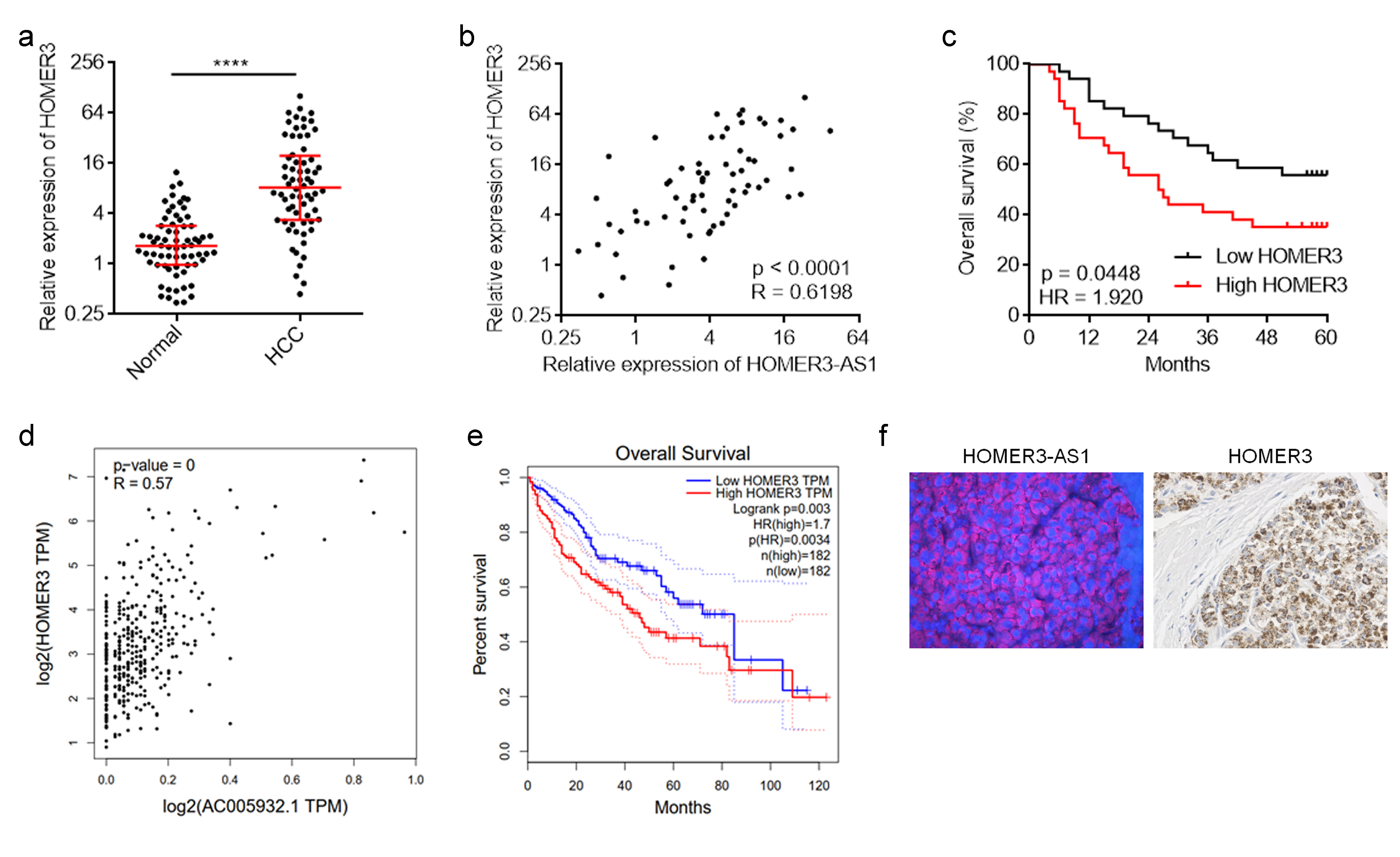

Supplement: Supplementary file 4 — Supplementary Figure 3 [file 41419_2021_4309_MOESM4_ESM.tif]

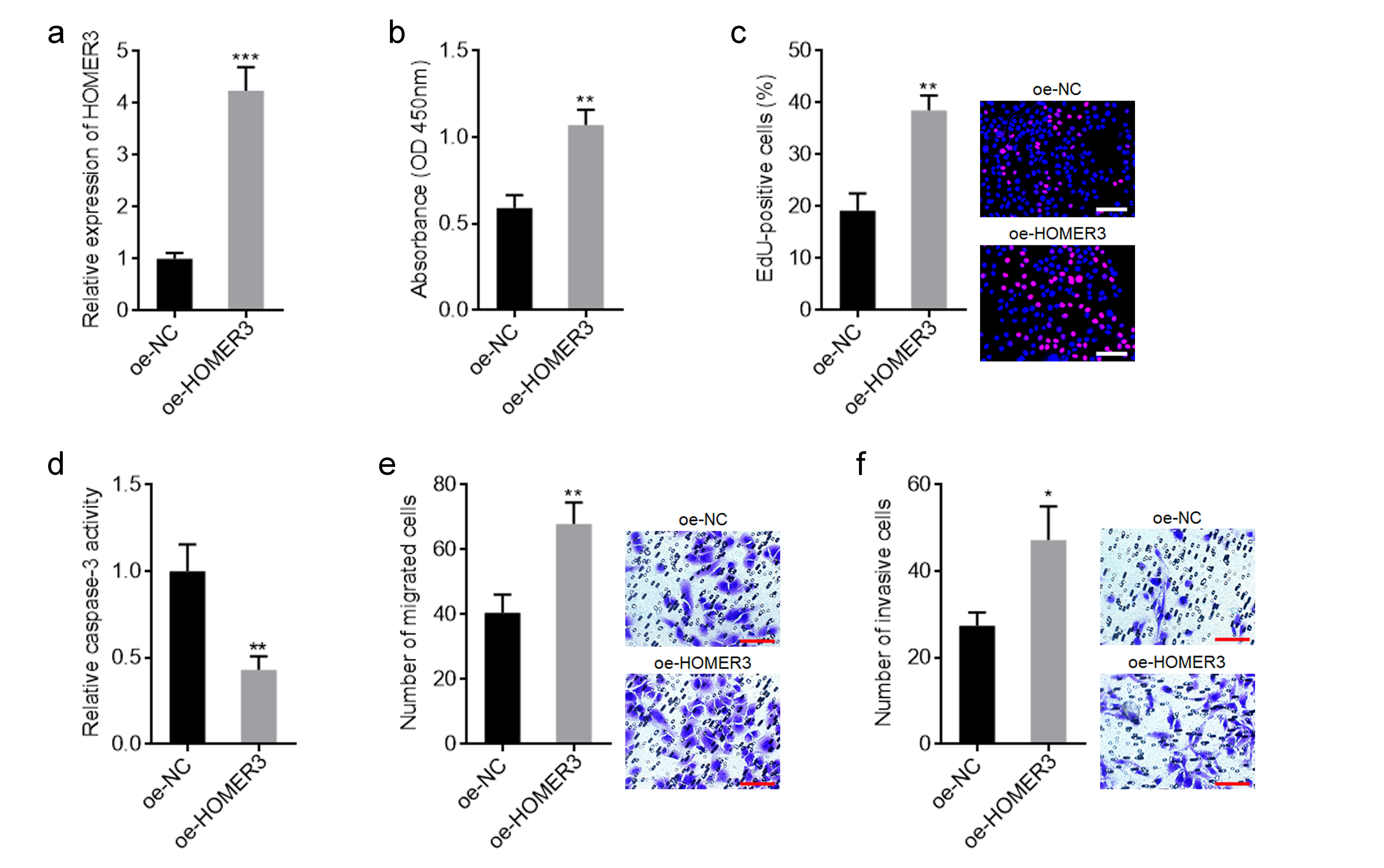

Supplement: Supplementary file 5 — Supplementary Figure 4 [file 41419_2021_4309_MOESM5_ESM.tif]

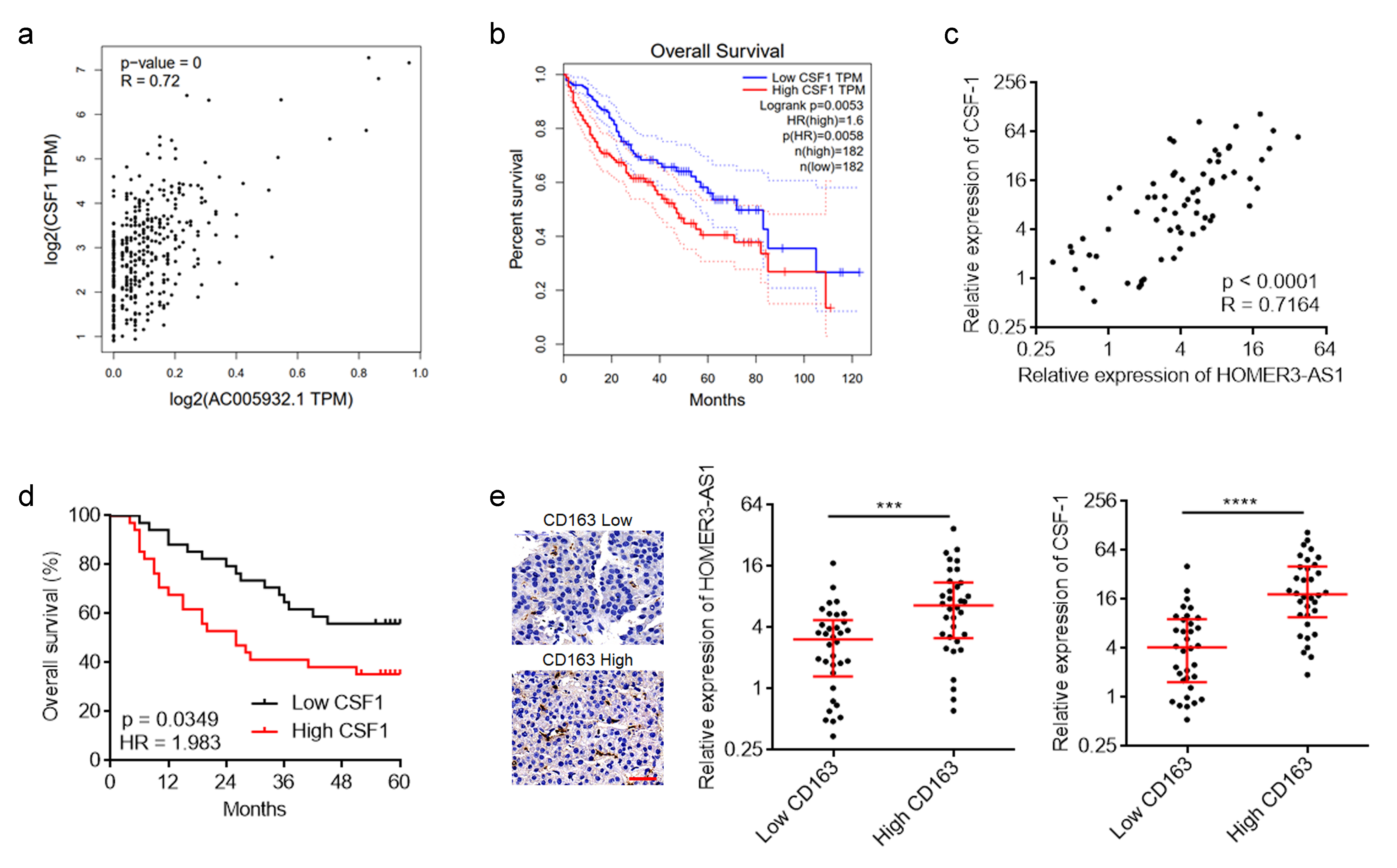

Supplement: Supplementary file 6 — Supplementary Figure 5 [file 41419_2021_4309_MOESM6_ESM.tif]
